# A simulation study of an electro-membrane extraction for enhancement of the ion transport via tailoring the electrostatic properties

**DOI:** 10.1038/s41598-022-16482-y

**Published:** 2022-07-16

**Authors:** Mahdiyeh Monesi, Mahdi Khatibi, Ahmad Rahbar-Kelishami

**Affiliations:** Research Lab for Advanced Separation Processes, Department of Chemical Engineering, Iran University of Science and Technology, Narmak, 16846-13114 Tehran Iran

**Keywords:** Chemical engineering, Pollution remediation

## Abstract

Membrane technology with advantages such as reduced energy consumption due to no phase change, low volume and high mass transfer, high separation efficiency for solution solutions, straightforward design of membranes, and ease of use on industrial scales are different from other separation methods. There are various methods such as liquid–liquid extraction, adsorption, precipitation, and membrane processes to separate contaminants from an aqueous solution. The liquid membrane technique provides a practical and straightforward separation method for metal ions as an advanced solvent extraction technique. Stabilized liquid membranes require less solvent consumption, lower cost, and more effortless mass transfer due to their thinner thickness than other liquid membrane techniques. The influence of the electrostatic properties, derived from the electrical field, on the ionic transport rate and extraction recovery, in flat sheet supported liquid membrane (FSLM) and electro flat sheet supported liquid membrane (EFSLM) were numerically investigated. Both FSLM and EFSLM modes of operation, in terms of implementing electrostatic, were considered. Through adopting a numerical approach, Poisson-Nernst-Planck, and Navier–Stokes equations were solved at unsteady-state conditions by considering different values of permittivity, diffusivity, and viscosity for the presence of electrical force and stirrer, respectively. The most important result of this study is that under similar conditions, by increasing the applied voltage, the extraction recovery increased. For instance, at EFSLM mode, by increasing the applied voltage from $$10 $$ to $$30 {\text{V}}$$, the extraction recovery increased from $$53$$ to $$98\%$$. Furthermore, it was also observed that the presence of nanoparticles has significant effects on the performance of the SLM system.

## Introduction

Nowadays, with the growth of technology, the amount of industrial wastewater discharged into the environment gradually increases. Even at low concentrations, contaminants in wastewater have devastating effects on human health and other living organisms. Metal ions are one of the most toxic pollutants in wastewater discharged into the environment^1–7^. Due to the widespread use of heavy metals such as cadmium in pigments, plating, metallurgy, and agricultural fields (fertilizers and pesticides), this toxic metal ion is released into water sources and contaminates them^8^. On the other hand, due to the lack of degradability and toxicity, the presence of these metals in water resources is very worrying for the ecosystem . For this reason, the World Health Organization (WHO) has defined $3{\text{ ppm}}$ as the maximum allowable concentration of cadmium in drinking water^9–11^. Therefore, it is necessary to develop effective and low-cost methods for removing metals from wastewater before disposal. There are various methods for removing metal ions from wastewater, such as liquid–liquid extraction^12–14^, adsorption^15,16^, ion exchange^17^, electrodialysis^18,19^, and membrane processes^2,20–22^.

Today, membrane technology has advantages such as reduced energy consumption due to no phase change, low volume and high mass transfer, high separation efficiency for dilute solutions, straightforward design of membranes, and ease of use on industrial scales from other methods^23^. Isolation agents are distinguished, among which the use of SLM contains two phases of the acceptor and donor due to high extraction efficiency even at low concentrations, low solvent consumption, low cost, more effortless mass transfer due to thinner thickness than Other LM techniques have received much attention^24^.

The SLM membrane can transmit the desired ion by the driving force of the voltage difference, or velocity. So far, many researchers in the field of modeling, simulation and experimentation have conducted numerous studies on liquid membranes for wastewater treatment, recovery of rare earth metals^25–29^. Tehrani et al.^30^ studied stabilized nanofluid membranes to separate gadolinium ions from nitrate solution medium. They investigated the effect of hydrophilic TiO_2_ and hydrophobic SiO_2_ nanoparticles on the stabilized liquid membrane system. The results showed that the presence of nanoparticles significantly affected the diffusion of the SLM system and concluded that hydrophobic nanoparticles are more desirable. Zaheri et al.^31^ recovered europium metal by carbon nanotubes and acid carriers (Cyanx 272) in the SLM system and investigated the effect of feed pH on separation quality. Bhatluri et al.^32^ investigated the removal of cadmium and lead from an aqueous feed by coconut oil as the solvent and Aliquate 336 as the carrier. By increasing EDTA to the receiving phase, they increased the mass transfer flux^33^. The separation of Cd (II) and Ni (II) ions in an aqueous sulfate medium using a stabilized liquid membrane (SLM) were studied. The effect of various parameters such as feed concentration, carrier concentration, feed phase, and receptor pH on the separation factor and flux of Cd (II) and Ni (II) ions was studied, which concluded that the percentage of cadmium separation is much higher than a nickel^34^. Rehman et al.^35^ investigated the transfer of zinc (II) through the SLM flat membrane with the carrier TDDA (tri-n-dodecylamine). The stoichiometry of the extracted species, i.e., complex, was investigated using slope analysis, and it was found that complex (LH)_2_·Zn(CL)_2_ is responsible for Zn (II) transmission. The predicted results of the mathematical model of zinc transfer (II) are consistent with the experimental results. Zn (II) flux was found to increase somewhat with increasing carrier and HCL in the feed solution and decreased with further increase of concentration. Martinez et al.^36^ studied the separation of Yttrium-Neodymium-Dysprosium mixture using bis (2-Ethylhexyl) hydrogen phosphate (D2EHPA) as a carrier by FSLM by simulation. Selectivity and a transient kinetic-infiltration model have been used in the calculations. The distribution of resistance between phases, pH, extractor concentration, and initial feed concentration dramatically affects the selectivity and process time, and their proper management improves the separation. The instability of the membrane phase affects the life of the membrane over time, which causes the organic phase of the membrane to disappear in the two aqueous phases. The phase turns blue and can destroy the separation unit.

Khosravikia et al.^37^, analyzed the transfer of acidic and alkaline drugs by EME and the effect of parameters such as applied voltage, membrane thickness, initial drug concentration, time, pH donor/ acceptor extraction pH, drug species penetration coefficient, and membrane porosity EME performance was evaluated by applying partitioning conditions. The most important conclusion of these studies is that flux was strongly dependent on SLM potential difference and increasing potential difference increased flux; the findings of this study can help understand the EME system better to find suitable conditions to increase EME extraction of the drug. Dolatabadi et al.^38^, investigated a binary numerical simulation to investigate the behavior of mass transfer and analyte retrieval in EME devices. The proposed model can describe the effect of different parameters on EME recovery. The predicted results show that the most critical factors in EME are analyte diffusion, analyte distribution coefficient, and the effective protonated surface in donor and acceptor solutions. The proposed model helps predict the mass transfer behavior of the EME process in practical applications. Chalik et al.^39^, examined the transfer of chromium (VI) ions using an EME electro-analytical approach using an SLM. In the EME-SLM process, the Danesi mass transfer model was used to calculate kinetic data, the velocity constant, flux, permeability, and recovery for each parameter studied. The proposed model investigated parameters such as carrier change, carrier concentration effects, and solvent change effect, and chromium (VI) transfer was obtained with a recovery of 54.73% in 100 min under optimal conditions.

Cadmium is a toxic heavy metal whose production increased during the twentieth century due to the production of nickel–cadmium batteries, metal cladding, and plastic stabilizers. Exposure to cadmium results from eating contaminated food, including leafy vegetables and grains, and drinking water or inhaling contaminated air. Intestinal absorption of cadmium is more excellent with iron, calcium, or zinc deficiency. Tobacco smoke is the most important source of cadmium exposure. Cadmium is effectively stored in organs, including the kidneys, liver, bones, lungs, central nervous system, and heart, and can therefore disrupt several biological systems^40^. In this study, ionic mass transfer and extraction recovery in SLM systems were thoroughly investigated. Using the numerical method (finite element), Poisson and modified Nernst-Planck, and Navier–Stokes (Stokes-Brinkmann) equations were simultaneously solved. The system in question was investigated for two different modes, i.e., EFSLM mode (electrical driving force is applied for ionic transport) and FSLM mode (velocity driving force is applied for ionic transport). The effects of the mentioned driving force, membrane thickness, porosity, strength, initial feed phase concentration, cadmium concentration on extraction recovery, and the mass transfer flux were investigated.

## Problem formulation

**Figure 1:**
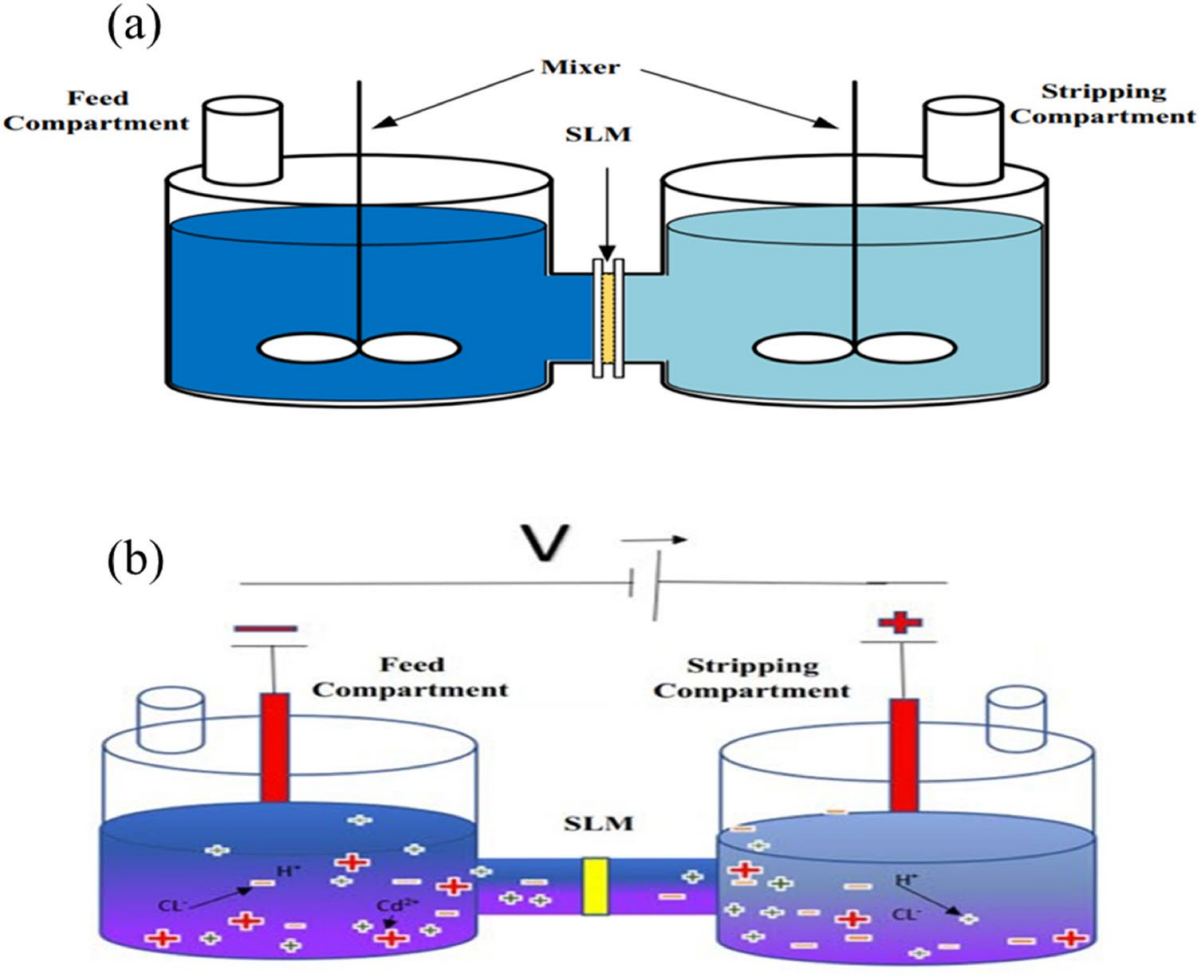
Schematic of the SLM process with the driving force, (**a**) velocity, and (**b**) electrical. (Note that panels (**a**) and (**b**) shows the FSLM, and EFSLM process, respectively).

**Figure 2:**
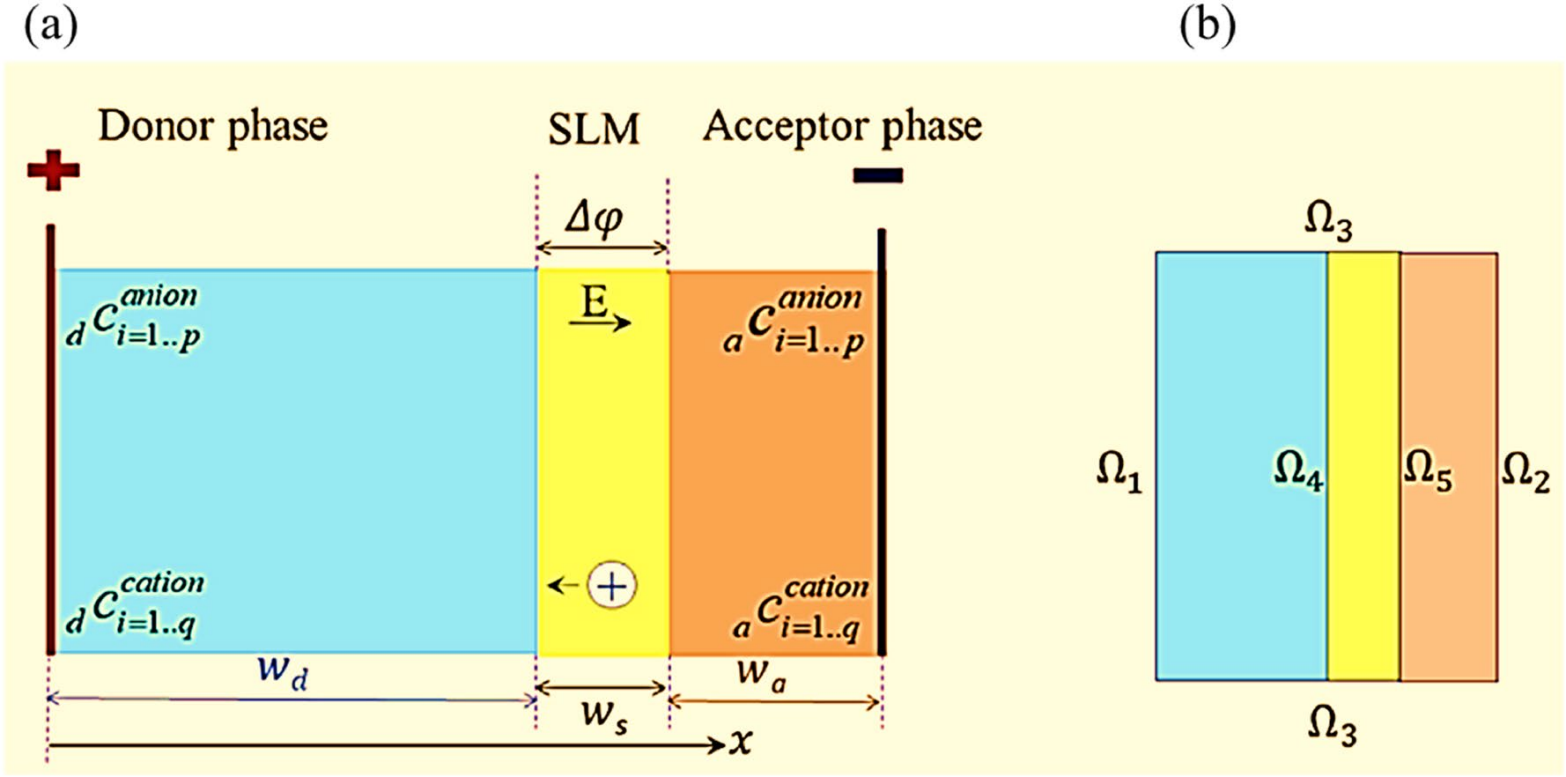
2-D view of the electromembrane extraction; (**a**) schematic of the equal setup of the system under study; (**b**) boundary conditions applied in operation.

As it is schematically shown in Fig. 1, ionic transfer has been applied to the liquid supported membrane in either of FSLM and EFSLM modes. The phenomena of ionic current rectification, ionic selectivity, and electroosmotic flow have occurred under unsteady-state conditions. As it can be seen in Fig. 2, the length and width of the reservoirs are equal to ${\text{H}}$,and ${\text{Wf}}$, respectively, and finally, the membrane thickness is equal to, ${\text{L}}_{{\text{m}}}$. Meanwhile, the reservoirs are large enough to ignore the end effects. In order to facilitate and reduce the computational workload, and regarding the symmetry of the chamber around its central axis, the calculations are conducted for half of the chamber. In case FSLM, the membran is located between two magnetic stirrer, Similarly, in EFSLM mode, the membrane is located between two electrodes, where the left electrode is the working electrode and the right electrode is connected to the ground. By applying a voltage, depending on its sign, an ionic current is established inside the channel. As it is shown in Fig. 2, for the EFSLM/FSLM, a cartesian coordinate system $\left( {{\text{x}},{\text{ y}},{\text{ z}}} \right)$ with its origin located on the SLM axis in the right tank wall is used^11,41^.

To solve the problem in question, it was assumed that the system is at unsteady-state condition and, as mentioned earlier, the flow regime is laminar (creeping flow), and the electrolyte is ${\text{HCl}}$ solution, which is a Newtonian and incompressible fluid. Meanwhile, it was considered; the fluid viscosity ${\upmu }_{{\text{E}}}$, diffusion coefficient of ionic species in the electrolyte ${\text{D}}_{{{\text{E}},{\text{j}}}}$ (${\text{j }} = { }1$ for cations $\left( {{\text{H}}^{ + } ,Cd^{ + 2} } \right)$ and ${\text{j }} = { }2$ For anions $\left( {{\text{Cl}}^{ - } } \right)$), the electrolyte permittivity ${\upvarepsilon }_{{\text{E}}}$, and ${\text{p}}$, ${\mathbf{u}}$, $\phi$, and ${\mathbf{N}}_{{\text{j}}}$, represent the hydrodynamic pressure, fluid velocity, electric potential, and flux of ionic species, respectively. The phenomenon is formulated using the modified Poisson-Nernst-Planck, and Navier–Stokes equations as follows:

In FSLM mode:

1 $$ \frac{{\partial {\mathbf{u}}}}{{\partial {\text{t}}}} + \mu_{{\text{E}}} \nabla^{2} {\mathbf{u}} - \nabla {\text{p}} = 0 $$

2 $$ \frac{{\partial (\varepsilon_{{\text{p}}} {\text{c}}_{{\text{i}}} )}}{{\partial {\text{t}}}} = - {\mathbf{u}} \cdot \nabla {\text{c}}_{i} + \nabla \cdot \left( { D_{E,i} \nabla {\text{c}}_{i} } \right) $$

In EFSLM mode:

3 $$ \varepsilon_{E} \nabla^{2} \phi = - \rho_{E} $$

4 $$ \nabla \cdot {\mathbf{N}}_{{\text{j}}} = \frac{{\partial {\text{c}}_{{\text{i}}} }}{{\partial {\text{t}}}} - \nabla \cdot \left( {D_{E,j} \nabla c_{j} + D_{E,j} \frac{{z_{j} Fc_{j} }}{RT}\nabla \phi } \right) = 0 $$

5 $$ \frac{{\partial (\varepsilon_{{\text{p}}} {\text{c}}_{{\text{i}}} )}}{{\partial {\text{t}}}} = - D_{E,i} \nabla {\text{c}}_{i} - \nabla \cdot \left( { D_{E,j} \frac{{z_{j} Fc_{j} }}{RT}\nabla \phi } \right) $$

In the above equations, the ionic density of the electrolyte is defined as ${\uprho }_{{\text{E}}} = \sum\nolimits_{{{\text{j}} = 1}}^{2} {{\text{z}}_{{\text{j}}} {\text{Fc}}_{{\text{j}}} }$ where ${\text{z}}_{{\text{j}}}$ and ${\text{c}}_{{\text{j}}}$ are the charge numbers and concentrations of ionic species in the electrolyte, respectively. The parameters ${\text{F}}$, ${\text{R}}$, ${\uprho }$ and ${\text{T}}$ denote the Faraday constant, the universal gas constant, fluid density, and the absolute temperature of the system, respectively.

Also, the extraction recovery percentage for each analyte is calculated according to the following:

6 $$ {\text{R}} = \frac{{{\text{n}}_{{{\text{a}},{\text{final}}}} }}{{{\text{n}}_{{{\text{s}},{\text{initial}}}} }} \times 100 = \frac{{{\oint }_{{V_{a} }} {\text{c}}_{{{\text{a}},{\text{final}}}} {\text{ dv}}}}{{{\oint }_{{V_{S} }} {\text{c}}_{{{\text{s}},{\text{initial}}}} {\text{ dv}}}} \times 100 $$

where $n_{{s,{\text{initial }}}}$ and ${\text{n}}_{{{\text{a}},{\text{final}}}}$ stand for the number of analyte moles initially available in the sample phase and finally in the acceptor phase, respectively. $V_{a}$ is the volume of acceptor phase, $V_{S}$ the sample volume, ${\text{c}}_{{{\text{a}},{\text{final}}}}$ the final concentration of analyte in the acceptor solution and ${\text{c}}_{{{\text{s}},{\text{initial}}}}$ is the initial analyte concentration in the donor solution.

The boundary conditions assumed for Eqs. (1)–(5) are given in Table 1 and shown in Fig. 2b.

**Table 1:** Boundary conditions considered for the computational regions illustrated in Fig. 2b.

Interfacial surfaces	Electrical potential	Ionic mass transfer	Flow field
${{\varvec{\Omega}}}_{1}$	Constant voltage bias $\phi = {\text{V}}_{{{\text{app}}}}$	ion-impervious ${\mathbf{n}} \cdot {\mathbf{N}}_{{\text{j}}} = 0$	No external pressure $p = 0$
${{\varvec{\Omega}}}_{2}$	Grounded $\phi = 0$	Ion-impervious ${\mathbf{n}} \cdot {\mathbf{N}}_{{\text{j}}} = 0$	No external pressure $p = 0$
${{\varvec{\Omega}}}_{3}$	Electrically neutral ${\mathbf{n}} \cdot \nabla \phi = 0 \,\,{\text{or}}\, \upsigma _{{\text{w}}} = 0$	Ion-impervious ${\mathbf{n}} \cdot {\mathbf{N}}_{{\text{j}}} = 0$	Slip boundary
${{\varvec{\Omega}}}_{4}$	Electrically neutral ${\mathbf{n}} \cdot \nabla \phi = 0\,\,{\text{ or}}\,\, \upsigma _{{\text{w}}} = 0$	Discontinuous at the SLM–liquid interface	Non-slip ${\mathbf{u}} = 0$
${{\varvec{\Omega}}}_{5}$	Electrically neutral ${\mathbf{n}} \cdot \nabla \phi = 0 \,\,{\text{or}}\, \upsigma _{{\text{w}}} = 0$	Discontinuous at the SLM–liquid interface	Non-slip ${\mathbf{u}} = 0$

## Solution method

**Figure 3:**
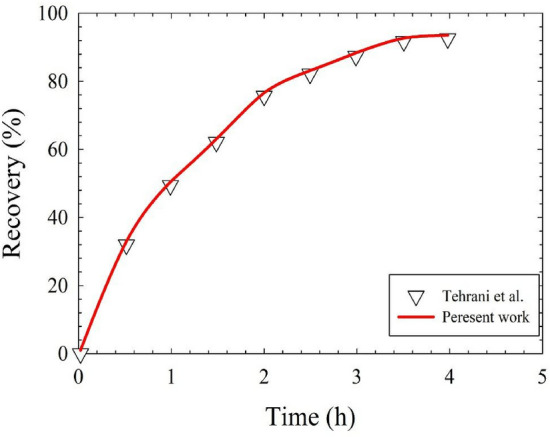
Comparison between numerical solution of the present model and experimental results of Tehrani et al.^42^.

Given that Eqs. (1)–(5) are interdependent and highly nonlinear; one should use appropriate numerical tools to solve them. Here, the equations were solved using Comsol Multiphysics software (5.6a), which works based on the high-performance finite element method. Electrostatics, transport of dilute species, and creeping flow physics were used to simulate the present study using a combination of triangular and square meshes. The mesh-independence study was performed on the conical geometry to determine the optimal mesh number. The results revealed that $81644$ meshes were sufficient for the FSLM/EFSLM as the optimal mesh number. In addition, to evaluate the performance of the present model, as you can see in Fig. 3, the results are successfully compared with the experimental data of Tehrani et al.^42^

.

## Results and discussions

The current research investigated the impacts of the different driving forces in both FSLM and EFSLM modes on ion transport and extraction recovery in a liquid-supported membrane. The influential variables that have been investigated include the driving force, membrane thickness, porosity, strength, initial feed phase concentration, cadmium concentration, and applied voltage. Values of parameters and variables used in the simulation process are given in Table 2.

**Table 2:** The values of parameters and constants employed in the simulations.

Parameter	Description	Value
$c_{0}$	Bulk concentration	$1, 10, 50, 100,200 {\text{mM}}$
$C_{d}$	Concentration of $Cd^{2 + }$	$1 \times 10^{ - 6} {-}1 \times 10^{ - 8} mM$
$D_{{E,k^{ + } }}$	Diffusivity of $K^{ + }$ in the electrolyte	$1.96 \times 10^{ - 9} {\text{m}}^{2} /{\text{s}}$
$D_{{E,Cl^{ - } }}$	Diffusivity of $Cl^{ - }$ in the electrolyte	$2.03 \times 10^{ - 9} {\text{m}}^{2} /{\text{s}}$
$D_{{E,Cd^{ + 2} }}$	Diffusivity of $Cd^{2 + }$ in the electrolyte	$9.38 \times 10^{ - 9} {\text{m}}^{2} /{\text{s}}$
$u_{0}$	Velocity, feed	$0.1 - 0.001 m/s$
$V_{app}$	Applied voltage	$10 - 30 V$
$e$	Elementary charge	$1.6022 \times 10^{ - 19} {\text{C}}$
$K_{B}$	Boltzmann constant	$1.381 \times 10^{ - 23} {\text{J}}/{\text{K}}$
$H$	Length of tank	$21 mm$
$L_{m}$	Thickness of membrane	$200 {\mu m}$
$W_{f}$	Width, phase feed and stripinng	$200 {\mu m}$
$r_{ - }$	The ionic radius of $Cl^{ - }$	$3.3 \times 10^{ - 10} {\text{ m}}$
$r_{ + }$	The ionic radius of $K^{ + }$	$3.3 \times 10^{ - 10} {\text{ m}}$
$k_{p}$	Permeability	$1 \times 10^{ - 8} - 1 \times 10^{ - 12} {\text{m}}^{2}$
$R_{S}$	Gas constant	$8.314\frac{J}{mol.K}$
$R_{T}$	Top radius	$10 {\text{nm}}$
$T$	Absolute temperature	$298.15 {\text{K}}$
$\varepsilon_{E}$	The relative permittivity of electrolyte	$80$
$\varepsilon_{0}$	Permittivity of vacuum	$8.854 \times 10^{ - 12} {\text{F}}/{\text{m}}$
$\varepsilon_{p}$	Porosity	$0.4 - 1$
$\rho$	Fluid density	$1000 {\text{kg}}/{\text{m}}^{3}$
$\mu_{E}$	Fluid viscosity within the electrolyte	$0.001 {\text{Pa}}.{\text{s}}$
$K$	Partition coefficient	$0.5 - 1$

### Effect of stirrer rate on ion separation rate

**Figure 4:**
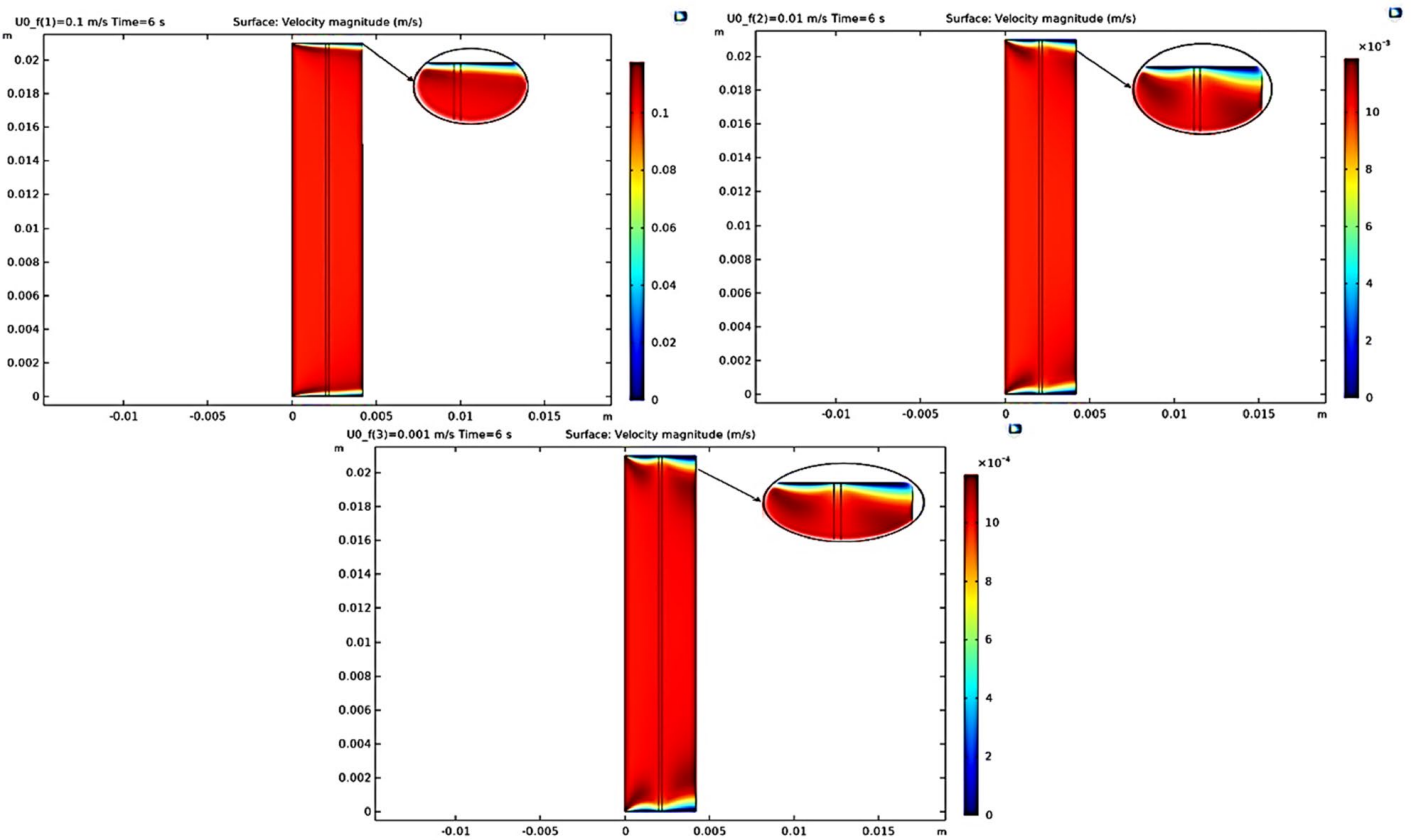
The effect of stirrer rate on ion separation rate in the FSLM system.

In order to present the results, we start by examining the ion separation contour in terms of different velocities at a fixed time of $6{\text{ S}}$ in the FSLM system by Fig. 4. As shown in Fig. 4, maximum separation is achieved in the middle region due to the turbulent flow created by the presence of the stirrer. In this area, the convective mass transfer increases, and the predominant mechanism in this area is eddy motion, and the separation rate increases for the two ends with a less turbulent diffusion mechanism. Also, increasing the velocity has a direct effect on the rate of separation.

**Figure 5:**
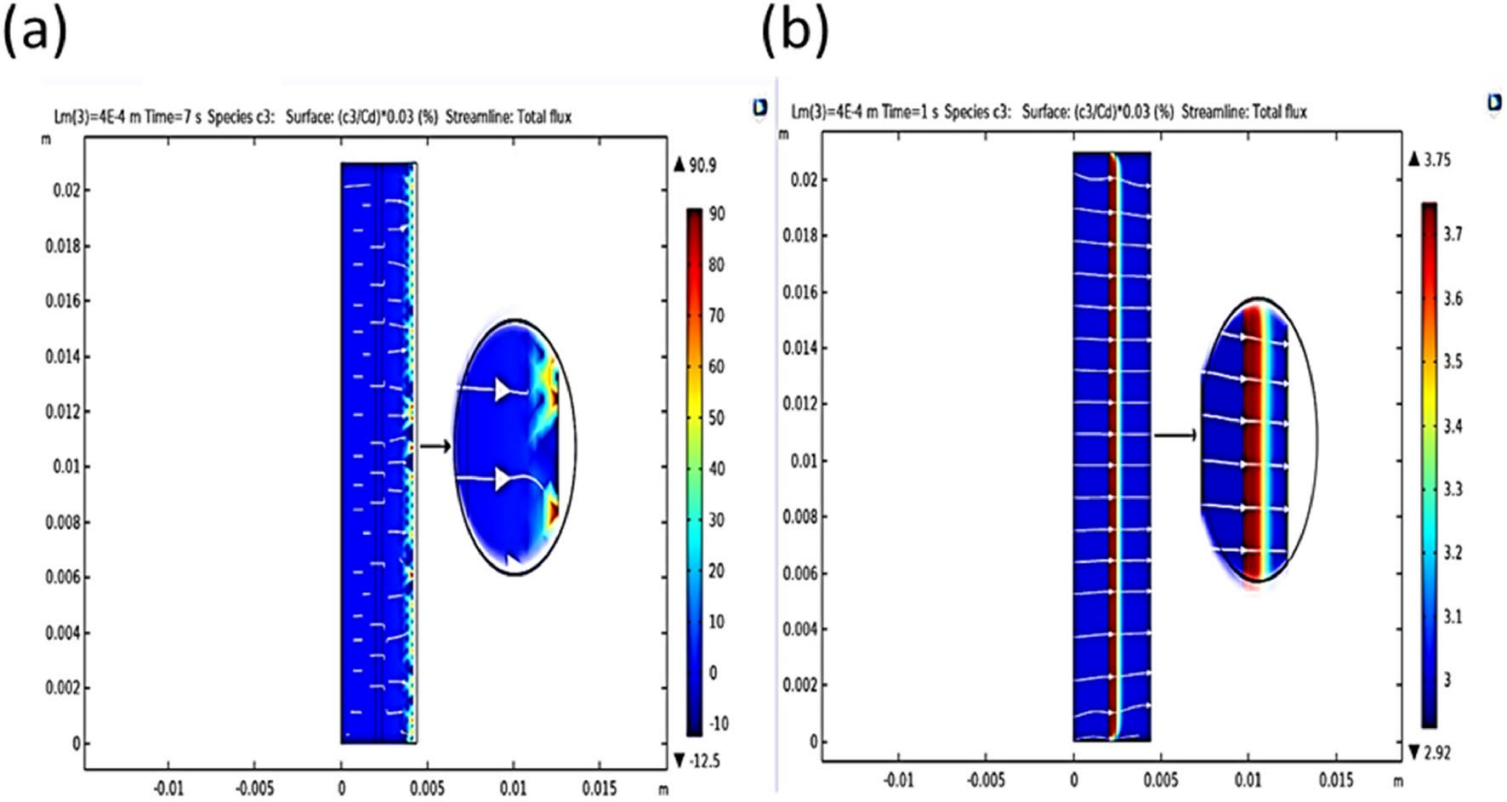
Comparison of the driving force, (**a**) electrical and (**b**) momentum on ion species transfer.

Figure 5 shows the effect of applied voltage and stirrer rate to transfer ionic species under $L_{m} = 200 \mu m$, $K = 0.8$, $c_{0} = 100 mM$, $\varepsilon_{p} = 0.8$, $u_{0} = 0.01m/s$, $V_{app} = 20 V$, and $k_{p} = 10^{ - 8} m^{2}$ conditions. As shown in Fig. 5, the transfer of ionic species in the EFSLM system is done with a uniform profile and plug. While in the FSLM system, the transfer of ionic species occurs in a dispersed and heterogeneous distribution. Because in the EFSLM system, due to the presence of electrostatic force and the formation of electrical double layers, the transfer of ionic species is faster and has a more significant effect on the separation efficiency^38^.

### Effect of velocity and voltage on ion separation rate

**Figure 6:**
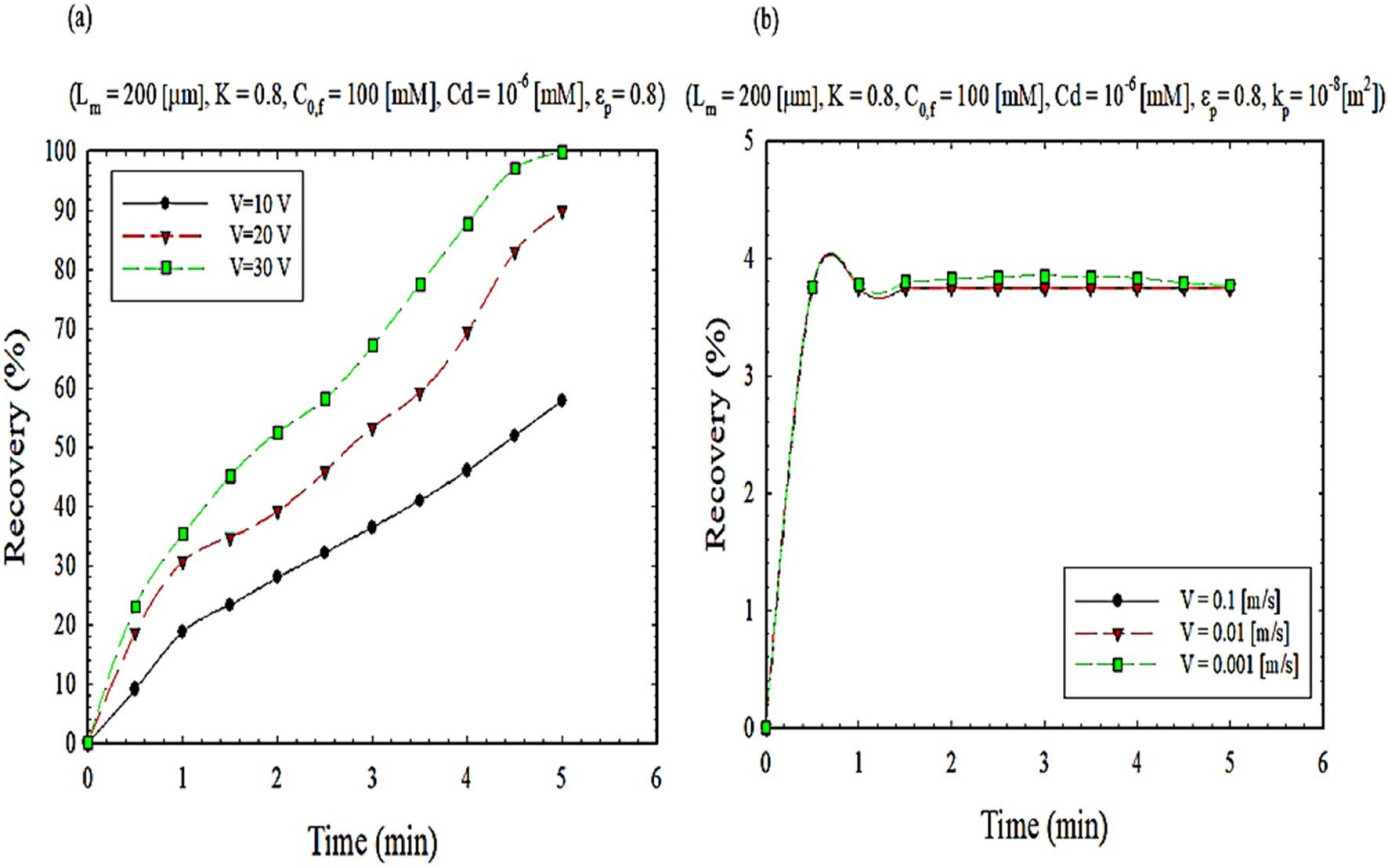
The impact of, (**a**) applied voltage, and (**b**) stirrer rate on extraction recovery.

The effect of velocity and voltage on ion separation is shown in Fig. 6. As shown in Fig. 6a, because the ion is inside the conductor, the applied voltage leads to electrophoretic motion and ion motion, whereas when velocity is the external factor, it will not have good logical outputs. As shown in Fig. 6a, increasing the voltage will directly affect the extraction efficiency. While shown in Fig. 6b, the membrane permeability is a function of the stirring speed on the solution side. By increasing the stirring speed too much, the permeability becomes independent of the stirring speed, so the separation, in this case, reaches its minimum value^43,44^.

### Effect of cadmium concentration on ion separation rate

**Figure 7:**
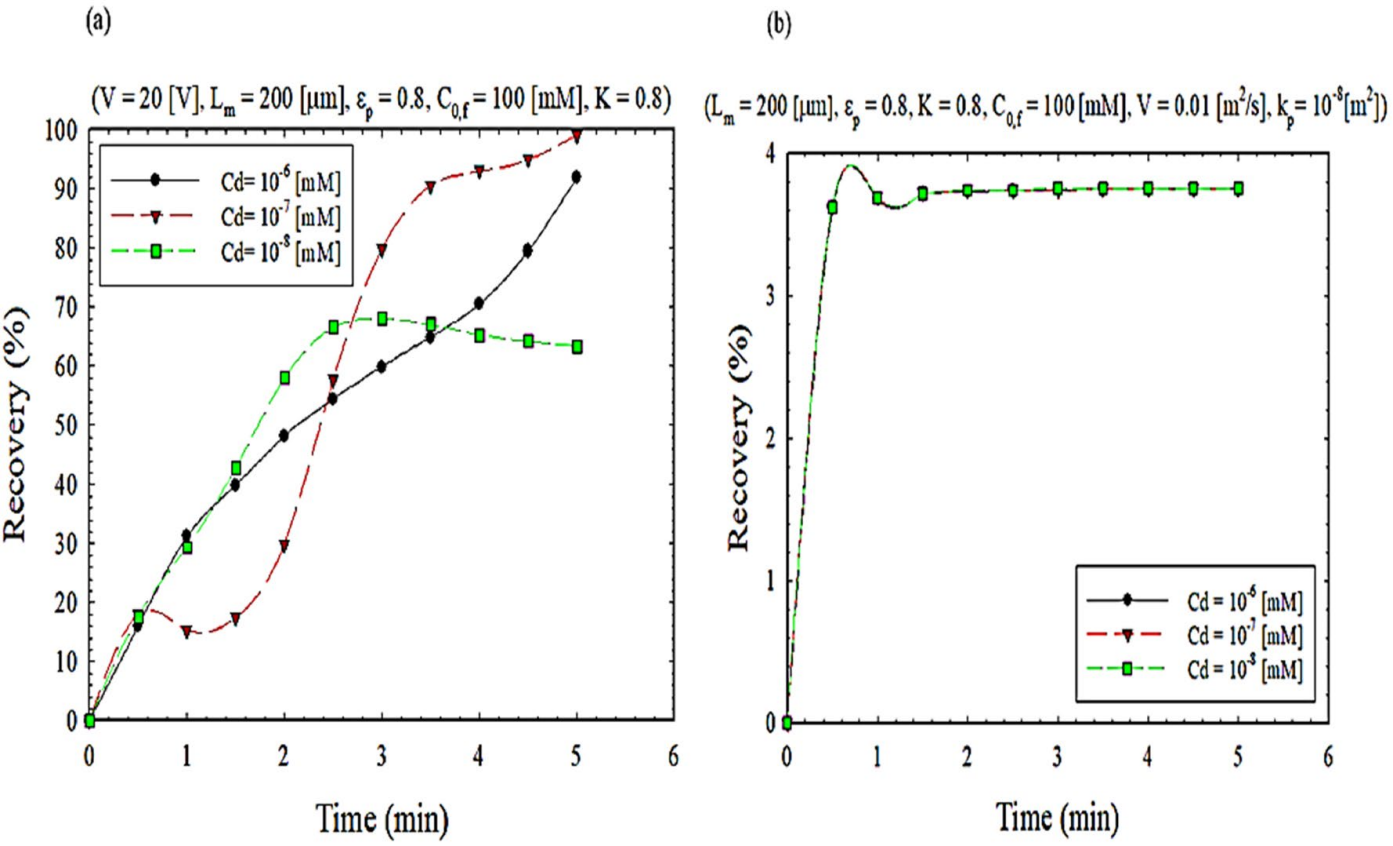
The impact of cadmium concentration on extraction recovery, (**a**) EFSLM, and (**b**) FSLM.

Figure 7 illustrates the effect of cadmium concentration on the separation rate. As shown in the electrostatic process, Fig. 7a, the mass transfer flux increases as cadmium (II) concentration increases. However, the percentage of cadmium ion extraction at higher metal ion concentrations is not significant because the organic phase is saturated with an ion-metal complex. Initially, at low concentrations of cadmium ions, the transfer of metal ions depends on the activity of the metal ions, which is the same concentration because the activity coefficient at low concentrations is one, but at high concentrations, the activity coefficient due to the Colombian interaction between anion and cation. Due to the increase in ionic strength, it leads to low salt activity and thus reduces extraction^45^.

### Effect of initial concentration on ion separation rate

**Figure 8:**
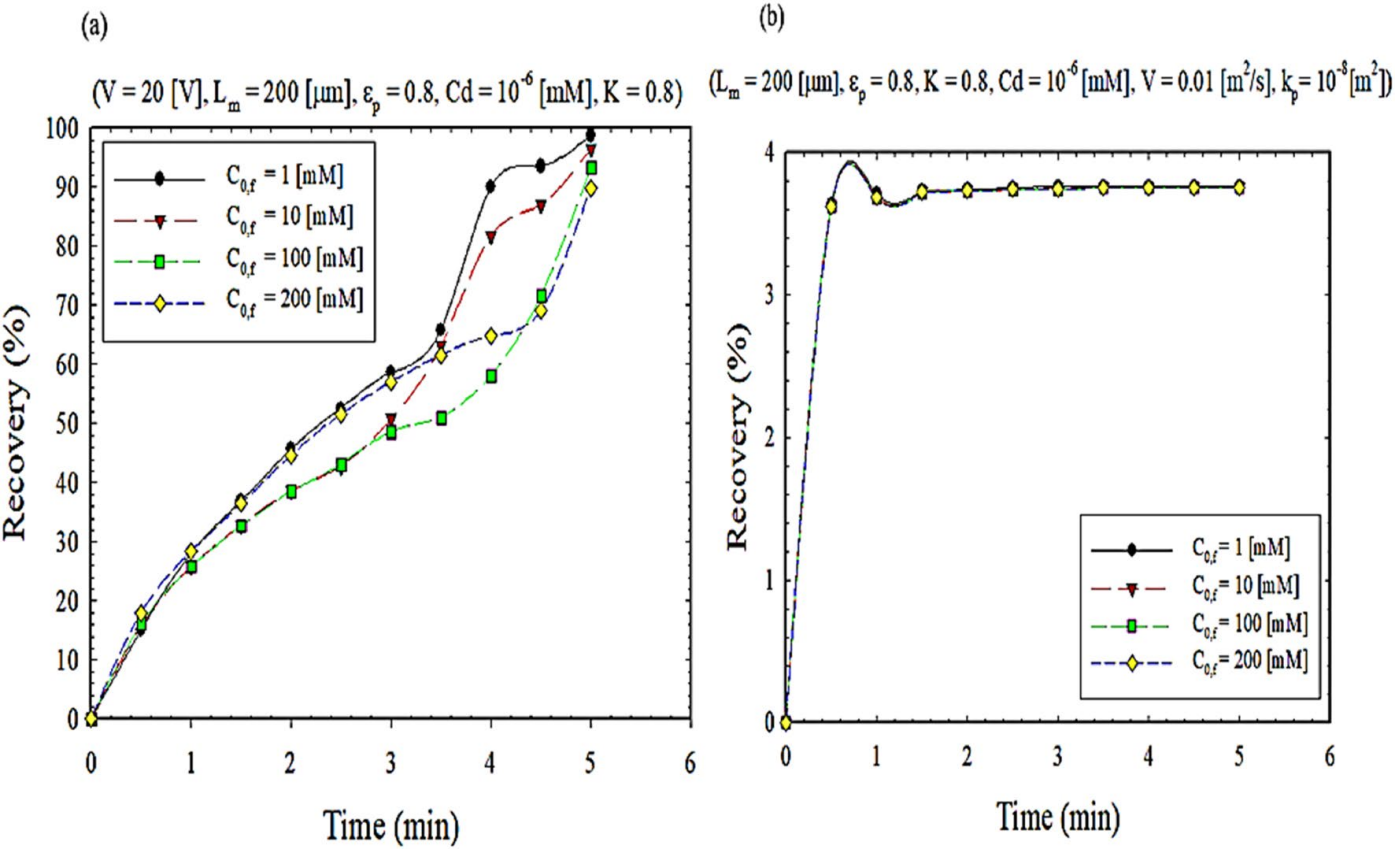
The impact of feed concentration on extraction recovery, (**a**) EFSLM, and (**b**) FSLM.

The effect of the initial concentration of the feed phase on the extraction efficiency at different times is shown in Fig. 8. As shown in Fig. 8a, as the initial concentration of the feed phase increases, the diffusion coefficient decreases, resulting in a mass transfer flux. Initially, as the cadmium concentration in the feed phase increases, the availability of cadmium ions to the feed-membrane boundary increases, leading to a faster increase in the surface chemical reaction and an increase in flux. This is due to the increase in the active site of mass transfer by filling the pores with complex-carrier species. However, after the membrane works for a long time, the pores of the membrane fill, and the separation decreases^46^.

### Effect of membrane properties on ion separation rate

**Figure 9:**
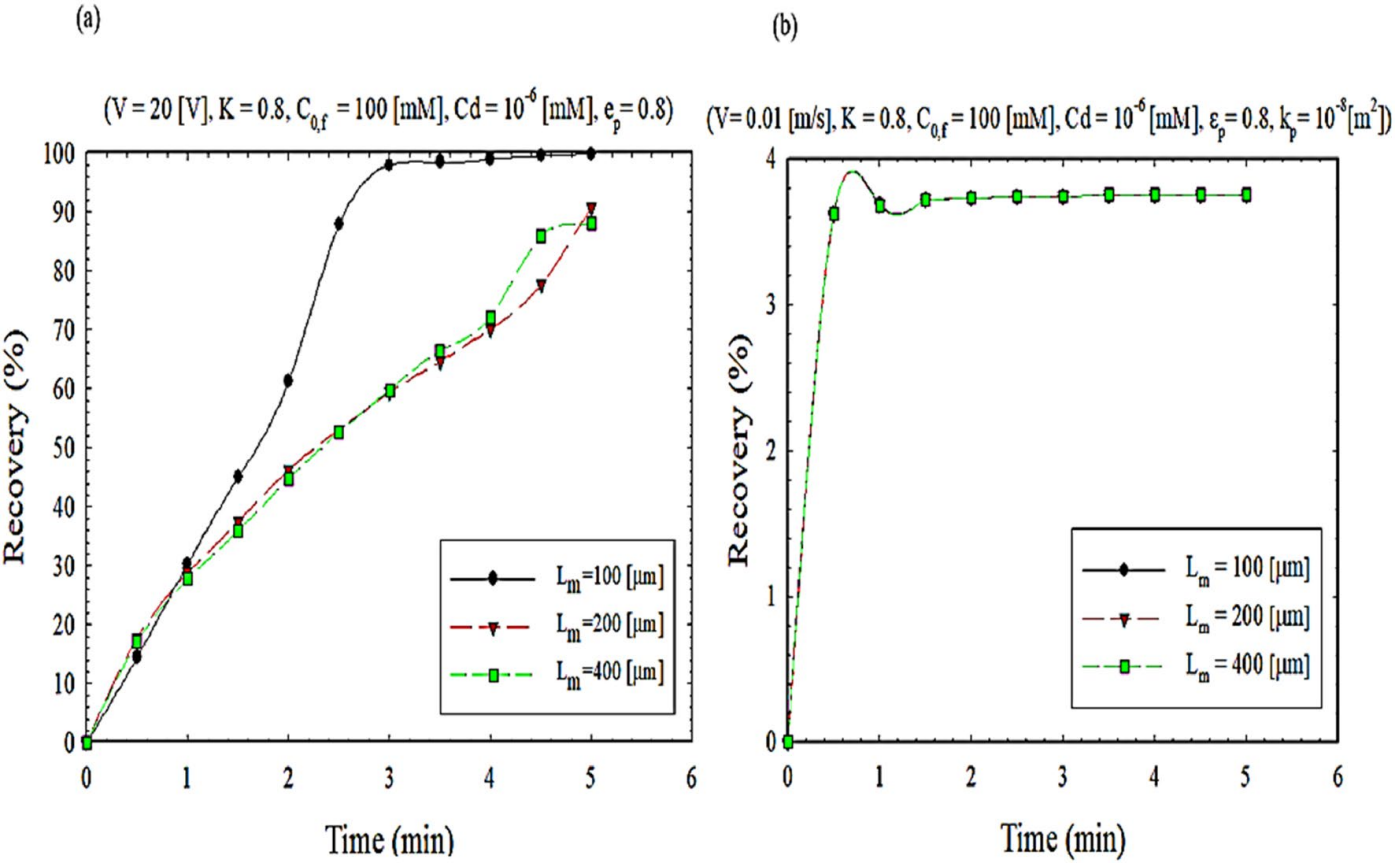
The impact of membrane thickness on extraction recovery, (**a**) EFSLM, and (**b**) FSLM.

**Figure 10:**
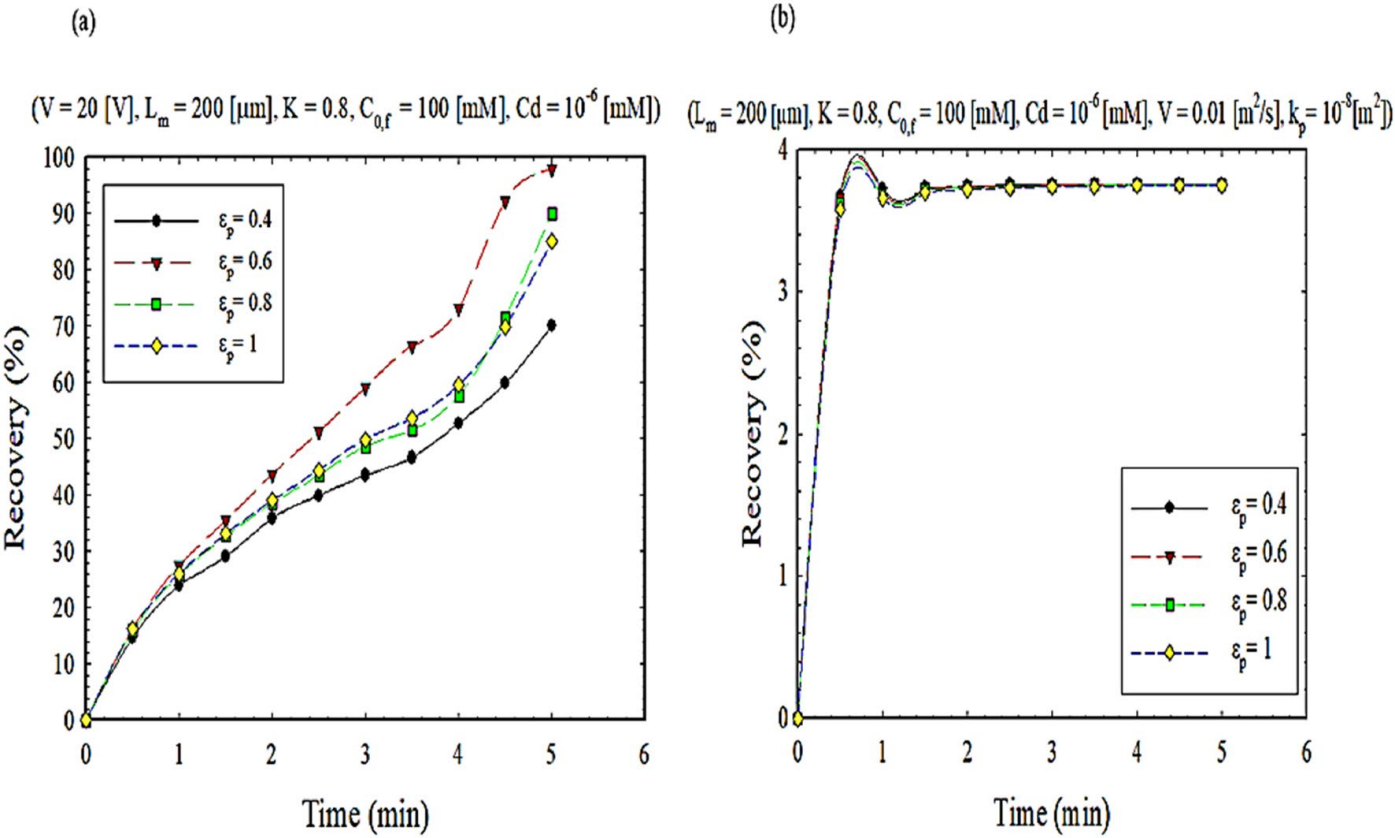
The impact of porosity on extraction recovery, (**a**) EFSLM, and (**b**) FSLM.

To achieve an exponential effect of the presence of nanoparticles in the present study on the separation rate, the membrane thickness distribution and porosity can be examined in Figs. 9 and 10, respectively, at different times. As shown in Fig. 9, the thickness of the membrane is proportional to the mass transfer flux. On the other hand, as the thickness of the membrane increases, the extraction percentage decreases, leading to a decrease in flux and permeability (Fig. S1). From another point of view, it can be said that the presence of nanoparticles increases the hydrophobic areas inside the membrane pores. It can also be concluded from Fig. 10 that increasing porosity increases permeability and further the separation of ions. On the other hand, there is no separation operation from a range onwards, leading to decreased separation efficiency (Fig. S2). In other words, the addition of nanoparticles reduces clogging and flux stability over a more extended period^47^.

### Effect of ionic partitioning on ion separation rate

**Figure 11:**
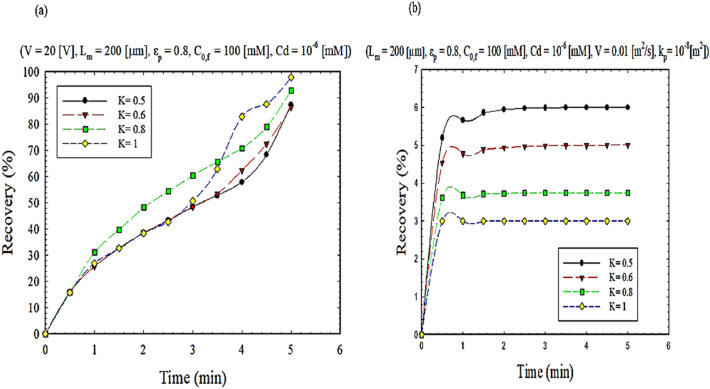
The impact of partition effect on extraction recovery, (**a**) EFSLM, and (**b**) FSLM.

As mentioned earlier, it was assumed that the mass transfer in the liquid membrane to separate the desired ion is accompanied by resistance. The effect of mass transfer resistance on extraction efficiency for two EFSLM systems, FSLM, is seen in Fig. 11. As can see in Fig. 11b. As k decreases, the recovery percentage increases. As k decreases, the space becomes smaller, and as you can see in the illustration, when it tilts towards 0.5, the ions tend to be inside an area with a more significant permeability. Yes, when the separation coefficient is applied, the permeability of one side is reduced. When the permeability is large, the concentration increases, and also, as shown in Fig. S3, k decreases, and as a result, the recovery percentage increases. While in Fig. 11a can see the opposite of this fact due to the creation of the force of the same name and repulsion between the loads, this reduces the recovery.

## Conclusions

The effect of the presence of electrostatic force and agitator by considering different values of permeability, the diffusion coefficient for the two systems EFSLM and FSLM, respectively, was studied in the present work. Unstable state simulations were performed using Poisson-Nernst-Planck and Laminar Flow equations using a finite element approach. The developed model was validated by comparing the results against existing theoretical and experimental data. The effect of different parameters (voltage, membrane thickness, porosity, resistance, feed phase initial concentration, cadmium concentration) on the separation rate in both FSLM and EFSLM modes has been investigated. Coincidentally, in all forms, the separation rate in the FSLM process using an agitator is much lower than in the EFSLM process using an electric field. The results have been evaluated by examining the effects of hydrodynamic parameters on separation performance over time. The effect of the presence of nanoparticles as surface activators for surface modification was also investigated, thereby preventing clogging, accumulation, and deposition as much as possible on the surface of the stabilized liquid membrane. The results show that the presence of nanoparticles has a significant effect on the penetration of the SLM system, and it can be concluded that hydrophobic nanoparticles are more desirable and affect the morphology of the membrane.

## Supplementary Information

Supplementary Information.

## Data Availability

Data are available [from Ahmad Rahbar-Kelishami] with the permission of [Ahmad Rahbar-Kelishami]. The data that support the findings of this study are available from the corresponding author, [Mahdiyeh Monesi], upon reasonable request.
