# Supplementary material for: A simulation study of an electro-membrane extraction for enhancement of the ion transport via tailoring the electrostatic properties
Source: Sci Rep. 2022 Jul 16;12:12170. doi: 10.1038/s41598-022-16482-y (PMC9288467; doi:10.1038/s41598-022-16482-y)
Supplement: Supplementary file 1 — Supplementary Information. [file 41598_2022_16482_MOESM1_ESM.docx]

**Supplementary Information**

**A simulation study of an electro-membrane extraction for enhancement of the ion transport via tailoring the electrostatic properties**

Mahdiyeh Monesi, Mahdi Khatibi, Ahmad Rahbar-Kelishami ^^[[1]](#footnote-1)^*^

*Research Lab for Advanced Separation Processes, Department of Chemical Engineering, Iran University of Science and Technolog, Narmak, Tehran 16846-13114, Iran*


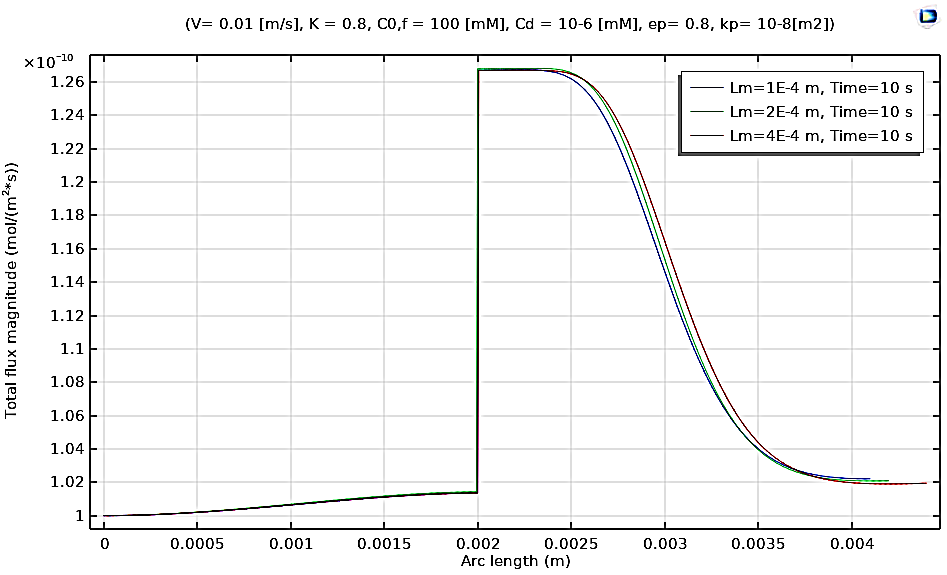


**Fig. S1.** The effect of total mass transfer flux along the membrane for different thicknesses in the FSLM system.


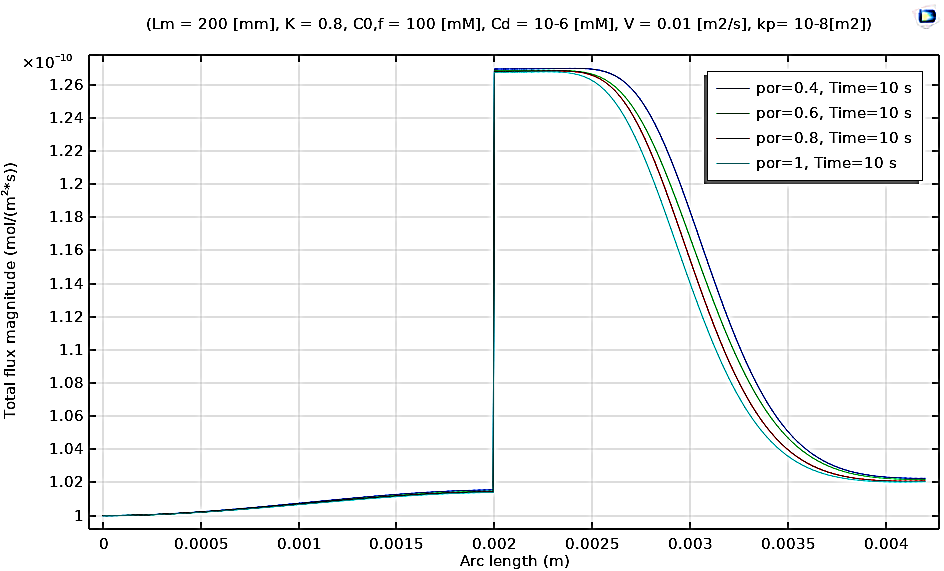


**Fig. S2.** The effect of total mass transfer flux along the membrane for different thicknesses in the EFSLM system.


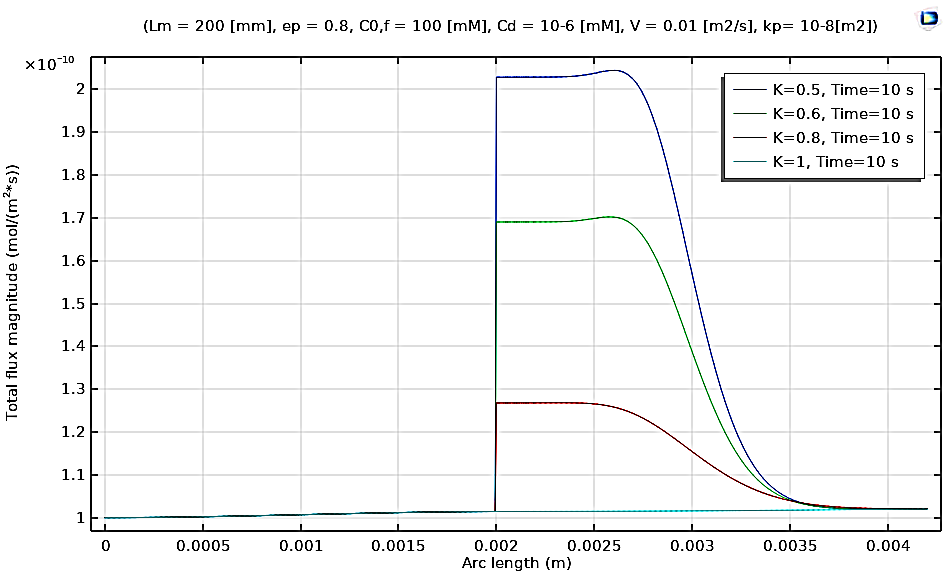


**Fig. S3.** The effect of total mass transfer flux along the membrane for different partition in the FSLM system.

1. [↑](#footnote-ref-1)
